# Comparative Analysis of *Peniophora lycii* and *Trametes hirsuta* Exoproteomes Demonstrates “Shades of Gray” in the Concept of White-Rotting Fungi

**DOI:** 10.3390/ijms231810322

**Published:** 2022-09-07

**Authors:** Alexander V. Shabaev, Konstantin V. Moiseenko, Olga A. Glazunova, Olga S. Savinova, Tatyana V. Fedorova

**Affiliations:** A.N. Bach Institute of Biochemistry, Research Center of Biotechnology, Russian Academy of Sciences, 119071 Moscow, Russia

**Keywords:** white-rot fungi, *Peniophora lycii* LE-BIN 2142, *Trametes hirsuta* LE-BIN 072, lignocellulose, secretome, exoproteome

## Abstract

White-rot basidiomycete fungi are a unique group of organisms that evolved an unprecedented arsenal of extracellular enzymes for an efficient degradation of all components of wood such as cellulose, hemicelluloses and lignin. The exoproteomes of white-rot fungi represent a natural enzymatic toolbox for white biotechnology. Currently, only exoproteomes of a narrow taxonomic group of white-rot fungi—fungi belonging to the Polyporales order—are extensively studied. In this article, two white-rot fungi, *Peniophora lycii* LE-BIN 2142 from the Russulales order and *Trametes hirsuta* LE-BIN 072 from the Polyporales order, were compared and contrasted in terms of their enzymatic machinery used for degradation of different types of wood substrates—alder, birch and pine sawdust. Our findings suggested that the studied fungi use extremely different enzymatic systems for the degradation of carbohydrates and lignin. While *T. hirsuta* LE-BIN 072 behaved as a typical white-rot fungus, *P. lycii* LE-BIN 2142 demonstrated substantial peculiarities. Instead of using cellulolytic and hemicellulolytic hydrolytic enzymes, *P. lycii* LE-BIN 2142 primarily relies on oxidative polysaccharide-degrading enzymes such as LPMO and GMC oxidoreductase. Moreover, exoproteomes of *P. lycii* LE-BIN 2142 completely lacked ligninolytic peroxidases, a well-known marker of white-rot fungi, but instead contained several laccase isozymes and previously uncharacterized FAD-binding domain-containing proteins.

## 1. Introduction

Wood-degrading basidiomycete fungi that cause white rot of the wood (i.e., white-rot fungi) are a unique group of organisms that evolved the ability to efficiently decompose all components of lignified plant cell walls [1]. Being able to release carbon stored in wood biomass, especially in such its recalcitrant component as lignin, these fungi play a fundamental role in carbon balance, soil formation and forest regeneration [2,3]. Currently, many scientists consider white-rot fungi as natural biochemical factories [4,5], the biotechnological use of which can not only improve the efficiency of enterprises associated with wood processing (i.e., biofuel and pulp and paper industries) [6,7], but also help to remove a wide range of recalcitrant soil and water contaminants, such as dyes, pesticides, industrial chemicals and pharmaceuticals [8,9,10].

Although a lot of information regarding the process of wood degradation by white-rot fungi has been assembled over the years, still there are many fundamental questions, one of which is the question of enzymatic machinery used by these fungi [11]. Currently, the main knowledge about enzymes secreted by white-rot fungi was obtained based on the fungi from Agaricales and Polyporales orders and there is a lack of information regarding white-rot fungi from other taxa [12]. Since the same changes in the wood appearance and chemical composition can potentially be achieved through the action of very different multi-component enzymatic systems, the more enzymatic machineries of white-rot fungi from different taxonomic categories and ecological niches are explored, compared and contrasted, the closer the scientific community will be to fully understand the great diversity of ways by which these fungi can challenge their substrates in order to satisfy their nutritional needs.

During our work, the comparison of two white-rot fungi *Peniophora lycii* LE-BIN 2142 and *Trametes hirsuta* LE-BIN 072 was performed. *T. hirsuta* LE-BIN 072 is a typical representative of the Polyporales order, more precisely of its Core Polyporoid clade [13]. This clade contains many white-rot fungi, such as *Trametes* spp., *Pycnoporus* spp. and *Polyporus* spp., that became model organisms for the white rot of wood [12], and more than 1000 articles have been published on different aspects of their biochemistry and molecular biology (PubMed search) [14]. At the same time, *P lycii* LE-BIN 2142 belongs to the well- supported Peniophoraceae clade of the recently elucidated order of homobasidiomycetes—Russulales [15]. Currently, fewer than 20 articles devoted to the biochemical aspects of these white-rot fungi have been published (PubMed search) [14], which makes them an excellent target for discovery of previously unnoticed aspects of the white rot type of wood degradation.

In this article, *P lycii* LE-BIN 2142 and *T. hirsuta* LE-BIN 072 were compared and contrasted in terms of their enzymatic machinery used for degradation of different types of wood substrates—alder, birch and pine sawdust. To the best of our knowledge, this is the third article reporting exoproteomes of a fungus from the *Peniophora* genus; the other two are Brenelli et al. [16] and Ma et al. [17]. Additionally, growth patterns of these fungi on various types of sawdust and on substrates that are the main components of plant cell walls (cellulose, xylan and lignin) were investigated.

## 2. Results

### 2.1. Characterization of Growth and Overall Oxidative and Cellulolytic Activities during the Cultivation on Solid Agar Media Containing Various Types of Sawdust

**Figure 1:**
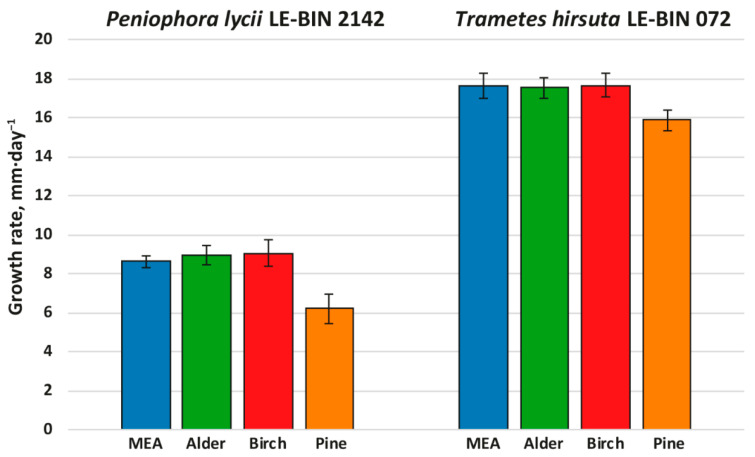
Growth rates of *P. lycii* LE-BIN 2142 and *T. hirsuta* LE-BIN 072 during their cultivation on MEA media containing various types of sawdust—alder, birch and pine. The MEA medium without sawdust addition was used as a control.

**Figure 2:**
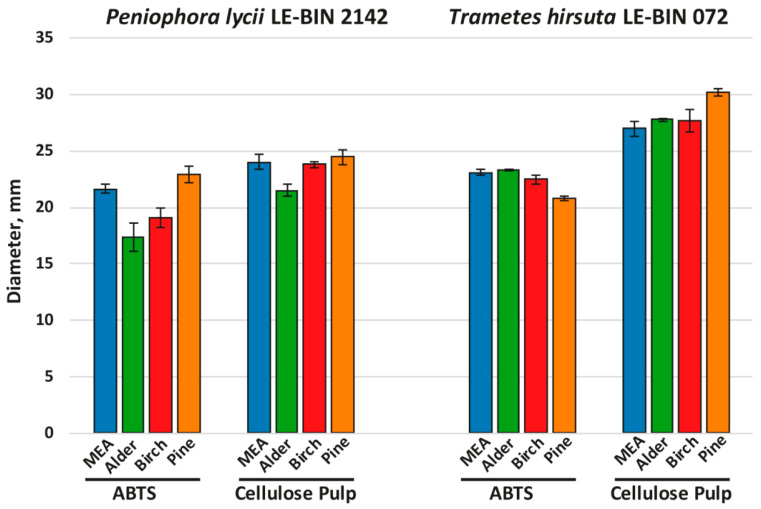
Results of agar plate assays of fungal overall oxidative (ABTS) and cellulolytic (cellulose pulp) activities.

To investigate the growth on different woody substrates, both *P. lycii* LE-BIN 2142 and *T. hirsuta* LE-BIN 072 were cultivated on solid agar media containing malt extract and various types of sawdust—alder, birch and pine; the medium containing only malt extract was used as a control. After the fungal mycelium had completely overgrown the Petri dish, fungal overall oxidative and cellulolytic activities were measured by agar plate assays with ABTS and cellulose pulp as substrates. The fungal growth rates are presented in Figure 1, and fungal overall oxidative and cellulolytic activities are presented in Figure 2.

Both *P. lycii* LE-BIN 2142 and *T. hirsuta* LE-BIN 072 were able to grow on all types of sawdust; however, their growth rates (Figure 1) and appearance of mycelium (Supplementary Figure S1) were significantly different. On all media, the growth rates of *P. lycii* LE-BIN 2142 were smaller than that of *T. hirsuta* LE-BIN 072. However, both fungi demonstrated the same tendency—the growth rates of each fungus on birch and alder sawdust were approximately the same and comparable to their growth rates on the control medium, while the growth rates on pine sawdust were significantly lower. In terms of overall appearance, on media with sawdust of birch and alder, the fungal mycelium was airier, and on pine it was denser and wadded. In addition, the mycelium of *P. lycii* LE-BIN 2142 differed in pigmentation on different media.

As can be seen from Figure 2 (and Supplementary Figure S1), the overall oxidative and cellulolytic activities of *P. lycii* LE-BIN 2142 were significantly lower than those of *T. hirsuta* LE-BIN 072 for almost all studied media and were more dependent on the medium type. For *P. lycii* LE-BIN 2142, the lowest overall oxidative activity was determined on the media with alder and birch sawdust, while the oxidative activities on the control medium and medium with pine sawdust were comparable. The overall cellulolytic activity of *P. lycii* LE-BIN 2142 was the lowest on the medium with alder sawdust and comparable between the control medium and media with birch and pine sawdust. For *T. hirsuta* LE-BIN 072, the overall oxidative and cellulolytic activities on the control medium and on the media with alder and birch sawdust were comparable. On the medium with pine sawdust, *T. hirsuta* LE-BIN 072 demonstrated the lowest oxidative and the highest cellulolytic activities.

### 2.2. Characterization of Growth and Overall Oxidative activity during Cultivation on Solid Agar Media Containing Different Wood-Derived Polysaccharides and Lignin

**Figure 3:**
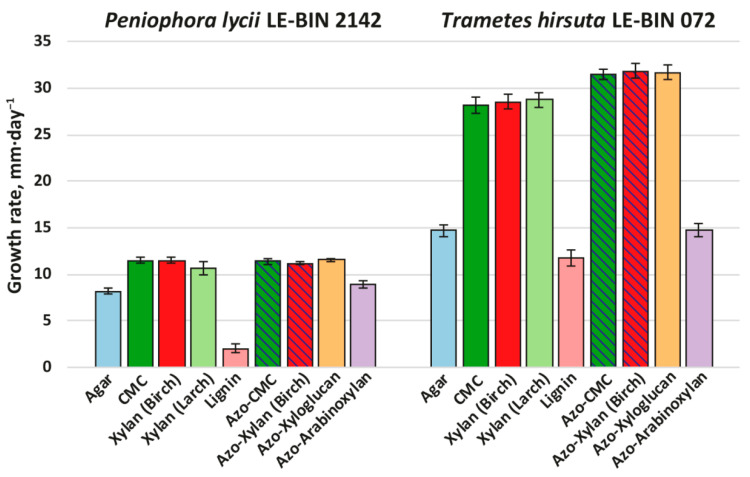
Growth rates of *P. lycii* LE-BIN 2142 and *T. hirsuta* LE-BIN 072 during their cultivation on solid agar media containing various wood components and their azo-labeled derivatives—CMC, birch xylan, larch xylan, lignin, azo-CMC, azo-birch xylan, azo-xyloglucan and azo-arabinoxylan. The control medium comprised agar as the only component. The azo-polysaccharide containing media for which counterparts without azo-labeling were tested are hatched.

To investigate the degradation ability toward different wood components, both *P. lycii* LE-BIN 2142 and *T. hirsuta* LE-BIN 072 were cultivated on solid agar plates containing either CMC, birch xylan, larch xylan, lignin, azo-CMC, azo-birch xylan, azo-xyloglucan or azo-arabinoxylan. The cultivation on medium composed of agar as the only component was used as a control. In the case of the media consisting of azo-polysaccharides, the additional information about overall oxidative activity was obtained based on the decolorization of these polysaccharides (more precisely, based on the decolorization of Remazol Brilliant Blue R (RBBR) dye, which is the coloring component of these polysaccharides) [18]. The fungal growth rates are presented in Figure 3.

Both *P. lycii* LE-BIN 2142 and *T. hirsuta* LE-BIN 072 were able to grow on all types of individual wood components. In comparison to their growth on various types of sawdust (see Section 2.1), both fungi formed thinner and less dense mycelial mats without aerial hyphae (i.e., produced less biomass per unit area). As on the sawdust, the growth rates of *P. lycii* LE-BIN 2142 were smaller than that of *T. hirsuta* LE-BIN 072 on all wood components. For both fungi, the growth rates on all polysaccharides were significantly higher than on the control medium containing only agar; the only exception was azo-arabinoxylan, on which the growth rate was comparable to that on the control medium. The growth of both *P. lycii* LE-BIN 2142 and *T. hirsuta* LE-BIN 072 was significantly inhibited by the presence of lignin.

The main differences in growth pattern of *P. lycii* LE-BIN 2142 and *T. hirsuta* LE-BIN 072 can be observed on the media containing unmodified and azo-modified polysaccharides (except azo-arabinoxylan). While for all these substrates growth rates of *P. lycii* LE-BIN 2142 were the same, the growth rates of *T. hirsuta* LE-BIN 072 were higher on azo-polysaccharides comparing with unmodified ones. Moreover, *P. lycii* LE-BIN 2142 did not decolorize azo-polysaccharides during its entire cultivation time (Supplementary Figure S2). In contrast, decolorization of azo-polysaccharides by *T. hirsuta* LE-BIN 072 correlated with its mycelial growth.

### 2.3. Characterization of Exoproteomes during Semi-Solid Cultivation on Media Containing Various Types of Sawdust

To identify the main enzymes involved in the processes of biodegradation of lignocellulosic material, both *P. lycii* LE-BIN 2142 and *T. hirsuta* LE-BIN 072 were cultivated by a semi-solid technique on **GP** media containing sawdust of alder (**GP+Alder**), birch (**GP+Birch**) and pine (**GP+Pine**). The cultivation on **GP** media without sawdust was used as a control. *P. lycii* LE-BIN 2142 was cultivated for 25 days, and *T. hirsuta* LE-BIN 072 was cultivated for 12 days. The longer cultivation time of *P. lycii* LE-BIN 2142 was chosen because of the twice lower growth rate demonstrated by this fungus (see Section 2.1 and Section 2.2).

**Figure 4:**
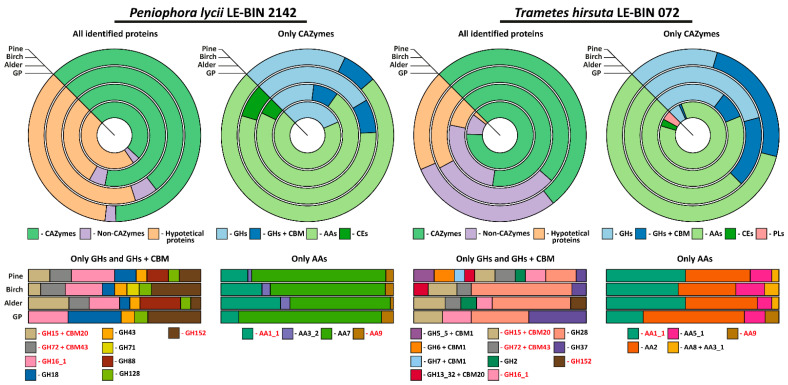
Approximate assessment of the relative quantities of the proteins detected in the exoproteomes of *P. lycii* LE-BIN 2142 and *T. hirsuta* LE-BIN 072 during their semi-solid cultivation on media containing various types of sawdust—alder, birch and pine. The CAZYmes secreted by both fungi are highlighted in red.

At the end of all cultivations, the culture liquids were collected and concentrated, after which proteins were precipitated and separated by the 2DE technique (Supplementary Figure S3). For each 2D gel, approximately 40 spots were analyzed by MALDI TOF/TOF MS/MS (Supplementary Table S2). As a result of matching of the proteins in analyzed spots with those annotated in the publicly available genomes, 41 and 48 different proteins were identified on all the cultivation media for *P. lycii* LE-BIN 2142 and *T. hirsuta* LE-BIN 072, respectively. The list of all the identified proteins, their detailed description and conditions under which they were detected are presented in Table 1, and the approximate assessment of the relative quantities is presented in Figure 4.

As is evident from Table 1 and Figure 4, the exoproteomes of *P. lycii* LE-BIN 2142 and *T. hirsuta* LE-BIN 072 differ both qualitatively and quantitatively. For the CAZyme proteins, only six out of twenty-four detected in all exoproteomes CAZyme families were secreted by both fungi, and patterns of their secretion were different. For the Non-CAZyme proteins, only S53-family proteases and putative isomerase YbhE were common for *P. lycii* LE-BIN 2142 and *T. hirsuta* LE-BIN 072 exoproteomes. Among hypothetical proteins secreted by both fungi, no homologues were found.

On all studied media, exoproteomes of both fungi were characterized by the presence of several majorly secreted proteins, and the identity of these proteins was different for *P. lycii* LE-BIN 2142 and *T. hirsuta* LE-BIN 072. For the *T. hirsuta* LE-BIN 072, the main proteins secreted on almost all media were laccases (CAZyme family AA1_1) and ligninolytic type II peroxidases (POD, CAZy family AA2). In contrast, all exoproteomes of *P. lycii* LE-BIN 2142 were characterized by the complete absence of peroxidases, and the main secreted protein was an FAD-binding domain-containing protein that was provisionally assigned to the AA7 CAZyme family.

Considering cellulose-acting enzymes, exoproteomes of *P. lycii* LE-BIN 2142 were characterized by the presence of lytic polysaccharide monooxygenase (LPMO, CAZyme family AA9) secreted on all media, GMC oxidoreductase secreted on the **GP+Alder, GP+Birch** and **GP+Pine** media, and an expansin-like protein secreted on **GP+Pine** medium. For *T. hirsuta* LE-BIN 072, almost all cellulose-acting enzymes (i.e., from CAZyme families GH5_5 + CBM1, GH7 + CBM1, GH6 + CBM1 and AA8 + AA3_1) were secreted only on **GP+Pine** medium; the exception was LPMO secreted only on **GP** medium and CDH secreted on all sawdust-containing media. Considering hemicellulose-acting enzymes, *P. lycii* LE-BIN 2142 secreted *α*-L-arabinofuranosidase/endo-*α*-L-arabinanase (CAZyme family GH43) during its growth on all sawdust containing media, and 4-O-methyl-glucuronoyl methylesterase (CAZyme family CE15) on the **GP+Alder** and **GP+Birch** media. *T. hirsuta* LE-BIN 072 secreted only one hemicellulase, *β*-mannosidase (CAZyme family GH2), during its growth on **GP+Alder** and **GP+Pine** media. Considering pectin-acting enzymes, while *P. lycii* LE-BIN 2142 secreted only GH88 on **GP+Alder** and **GP+Pine** media, *T. hirsuta* LE-BIN 072 secreted GH28 on all studied media and CE1 on **GP** medium. Considering starch-acting enzymes, both fungi secreted glucoamylases (CAZyme family GH15 + CBM20)—*P. lycii* LE-BIN 2142 on **GP+Alder**, **GP+Birch** and **GP+Pine** media and *T. hirsuta* LE-BIN 072 on all media. Additionally, the secretion of maltogenic amylase (CAZyme family GH13_32 + CBM20) was detected for *T. hirsuta* LE-BIN 072 on **GP+Birch** and **GP+Pine** media. Considering lignin-acting enzymes, as it was already mentioned, the main ligninolytic enzymes of *T. hirsuta* LE-BIN 072 were peroxidases and to a lesser extend laccases, and the main ligninolytic enzymes of *P. lycii* LE-BIN 2142 were presumably FAD-binding domain-containing proteins and to a lesser extent laccases.

In addition to plant cell wall-degrading enzymes, both *P. lycii* LE-BIN 2142 and *T. hirsuta* LE-BIN 072 secreted enzymes targeting components of fungal cell walls, proteases and proteins with unknown functions. In the case of enzymes targeting fungal cell walls, on each medium both fungi secreted at least one of the following enzymes: endo-b-1,3(4)-glucanases/lichenase-laminarinases (CAZyme family GH16_1), *β*-1,3-glucanase (CAZyme family GH152) and *β*-1,3-glucanosyltransglycosylase (CAZyme family GH72 + CBM43). In the case of proteases, *P. lycii* LE-BIN 2142 secreted only one protease from S53 family on all media. In the exoproteomes of *T. hirsuta* LE-BIN 072 three proteases from S10 and S53 families were detected on all studied media; metallopeptidase M36 was detected on **GP+Alder** and **GP+Pine** media; peptidase S8 (S08A) was detected on **GP**, **GP+Alder** and **GP+Pine** media; and two proteases from A01A family were detected on **GP+Birch** and **GP+Pine** media. In the case of proteins with unknown functions, it should be noted that for both fungi these proteins were relatively short; their median length comprised 195 and 166 amino acids for *P. lycii* LE-BIN 2142 and *T. hirsuta* LE-BIN 072, respectively.

## 3. Discussion

### 3.1. General Comparison of P. lycii LE-BIN 2142 and T. hirsuta LE-BIN 072

Comparison of the white-rot fungus *P. lycii* LE-BIN 2142 belonging to the poorly studied Russulales order with the typical white-rot fungus *T. hirsuta* LE-BIN 072 from the Polyporales order demonstrated interesting features of the former. Although many characteristics of *P. lycii* LE-BIN 2142 were similar to that of *T. hirsuta* LE-BIN 072, reflecting the common white-rot nature of both fungi, *P. lycii* LE-BIN 2142 also demonstrated substantial peculiarities.

During all solid-state cultivation experiments, although *P. lycii* LE-BIN 2142 demonstrated significantly smaller growth rates on all studied media, its growth patterns were generally similar to that of *T. hirsuta* LE-BIN 072. Reflecting that both *P. lycii* LE-BIN 2142 and *T. hirsuta* LE-BIN 072 belong to the class of white-rot fungi, which typically grow on the wood of deciduous trees, their growth rate was suppressed by the presence of pine sawdust and arabinoxylan, which is an abundant component of cell walls of conifers and grasses [22]. Furthermore, as white-rot fungi both *P. lycii* LE-BIN 2142 and *T. hirsuta* LE-BIN 072 were able to grow, though slowly, on kraft lignin. The main difference between *P. lycii* LE-BIN 2142 and *T. hirsuta* LE-BIN 072 was observed for azo-polysaccharides. The growth of *T. hirsuta* LE-BIN 072 on azo-polysaccharides was accompanied by their discoloration. In contrast, *P. lycii* LE-BIN 2142 did not decolorize azo-polysaccharides, although it grew steadily on them, which is atypical for white-rot fungi. Previously, in the analysis of 80 fungal strains corresponding to 52 species from 48 genera, and 22 families (plus 2 *incertae sedis*) [18], it was shown that almost all white-rot fungi were able to decolorize azo-polysaccharides.

**Figure 5:**
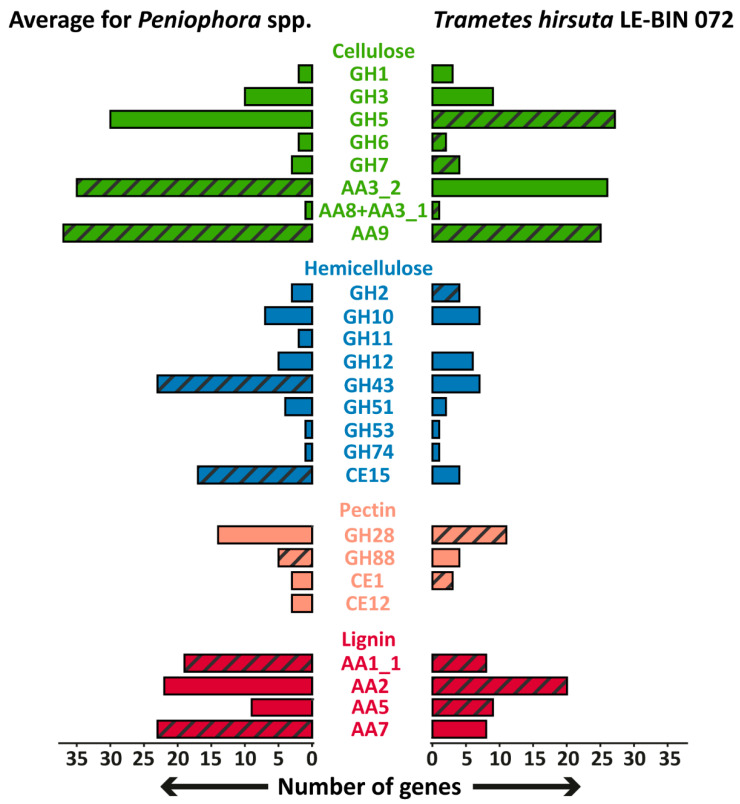
The comparison of the genome contents of *Peniophora* spp. and *T. hirsuta* LE-BIN 072. Groups of CAZymes that were detected in exoproteomes are hatched. The genes encoding CAZymes from GH26 and GH62 families were absent in the genomes of all fungi.

Unfortunately, direct comparisons of the *P. lycii* LE-BIN 2142 genome with that of other white-rot fungi is impossible since the genome of this fungus was not sequenced. However, we collected all the available genomes of fungi from the genus *Peniophora* and analyzed their genome content with respect to the genes encoding main wood-degrading enzymes [23]. The comparison of the genome contents of *Peniophora* spp. and *T. hirsuta* LE-BIN 072, as a typical representative of white-rot fungi, is shown in Figure 5. In general, the genome contents of *Peniophora* spp. and *T. hirsuta* LE-BIN 072 are typical for white-rot fungi. Both these genomes contain genes of CAZymes from the GH6, GH7, CE1 and AA2 families and CDH (AA8 + AA3_1), the presence of which are highly correlated with white-rot phenotype [24,25]. The main peculiarity of the *Peniophora* spp. genomes is an extreme expansion of genes encoding CAZymes from the AA9, AA7 and AA1_1 families, which is abnormal for typical white-rot fungi from Agaricales and Polyporales orders [24].

### 3.2. Secretion of Enzymes Related to Degradation of Wood Polysaccharides: Cellulose, Hemicelluloses and Pectin

For the degradation of polysaccharides, two main interconnected routes which can be used by wood-degrading fungi were previously described [23]: the long-known classical hydrolytic route and the recently discovered oxidative route. By the classical hydrolytic route, cellulose fibers firstly hydrolytically cleaved down to cellobiose by the mixture of endocellulases of the GH5 CAZyme family and exocellulases of the GH6 and GH7 CAZyme families, and thereafter cellobiose is hydrolyzed to glucose by cellobiases of the GH1 and GH3 CAZyme families. Hydrolytic cleavage of hemicelluloses and pectin are performed by CAZymes from various families: CAZymes from the GH10 and 11 families cleaved xylan; from GH2, 5 and 26—galactomannan; from GH12 and 74—xyloglucan; from GH62—arabinoxylan; from GH43—xylan, xyloglucan and pectin; and from GH28—pectin.

The discovery of the oxidative route of polysaccharide degradation is closely linked with the discovery of LPMO in 2010–2011 [26]. Cellulolytic LPMO is a copper-containing monooxygenase of the AA9 CAZyme family that catalyzes direct oxidative cleavage of the cellulose chain at random internal positions [27]. The LPMO reactions can be “fueled” by a variety of small molecular weight-reducing agents (e.g., ascorbic acid, phenols, lignin and lignin fragments) and enzymes capable of delivering reducing equivalents, the best-known example of which are FAD-domain-containing enzymes belonging to the GMC superfamily of oxidoreductases (CAZyme family AA3), especially cellobiose dehydrogenases (CDH, CAZyme family AA8+AA3_1). In addition to cellulose degradation, the enzymatic oxidative cleavage of hemicelluloses by the coordinated action of LPMO and CDH was recently confirmed [27].

Regarding the hydrolytic route of polysaccharide degradation, the exoproteomes of *P. lycii* LE-BIN 2142 and *T. hirsuta* LE-BIN 072 were relatively poor. Both fungi did not secrete cellobiases, and endo- and exocellulases were secreted only by *T. hirsuta* LE-BIN 072 on the medium containing pine sawdust. The only hemicellulase that was detected in the exoproteome of *P. lycii* LE-BIN 2142 belonged to the GH43 family, which includes many enzymes involved in hemicellulose and pectin debranching and degradation, and the only hemicellulase that was detected in the exoproteome of *T. hirsuta* LE-BIN 072 was β-mannosidase (CAZyme family GH2), which releases mannose from the non-reducing ends of galactomannans. Additionally, during cultivation on birch and alder sawdust, *P. lycii* LE-BIN 2142 secreted 4-O-methyl-glucuronoyl methylesterase (CAZyme family CE15), capable to hydrolyze lignin–carbohydrate esters.

Regarding the oxidative route of polysaccharide degradation, our data suggest that this is the main route used by *P. lycii* LE-BIN 2142. LPMO was identified in all exoproteomes of *P. lycii* LE-BIN 2142, and GMC oxidoreductase of the AA3_2 CAZyme family was identified on all sawdust-containing media. In the exoproteomes of *T. hirsuta* LE-BIN 072, although CDH was secreted on all sawdust-containing media, LPMO was identified only on the control GP medium.

Comparing our data on the exoproteome of *P. lycii* LE-BIN 2142 with the only two previously published exoproteomes of *Peniophora* spp., it can be proposed that the oxidative route of carbohydrate degradation can be typical for this fungal genus. In the exoproteome of *Peniophora* sp. CBMAI 1063 cultivated in a bioreactor on a nutritionally rich medium supplemented with CuSO_4_, although cellulases of GH3 and 5 CAZyme families were detected, their relative abundances (measured as spectral count) were significantly lower than that of LPMO and GMC oxidoreductases [16]. Similarly, while LPMO and GMC oxidoreductases were detected in the exoproteome of *Peniophora incarnata* T-7 grown in poplar wood-containing media, typical cellulases such as endoglucanases and exoglucanases were absent [17]. Regarding hemicellulases, the exoproteome of *Peniophora* sp. CBMAI 1063 was also poor in this type of enzyme [16]. *P. incarnata* T-7 secreted many hemicellulases during its growth on poplar wood, although, oddly enough, the same hemicellulases but in smaller amounts were produced on a control medium containing glucose as the only carbon source [17].

For the *T. hirsuta* LE-BIN 072, the poor secretion of cellulose- and hemicellulose-degrading enzymes on sawdust-containing media (with an exception of **GP+Pine** medium) was unexpected. Previously reported cultivations of fungi from the Polyporaceae family, specifically from *Polyporus* [28], *Pycnoporus* [29,30,31], *Dichomitus* [32] and *Trametes* [33,34,35] genera, demonstrated that these fungi almost always secreted quite a large number of typical cellulases and hemicellulases upon an induction by sawdust from various woody plants. The difference between our data and those published earlier can be explained by such uncontrollable experimental factors as decay stage of woody substrate with which fungal mycelium directly interacts. Recently performed experiments with *Trametes versicolor* cultivated on aspen wood wafers demonstrated absence of transcription of the genes encoding cellulases and hemicellulases and upregulation of pectinase encoding genes at the early decay stages; in contrast, upregulation of cellulases and hemicellulases together with absence of transcription of pectinases were observed at the late decay stages [36].

In summary, at least on **GP+Pine** medium, *T. hirsuta* LE-BIN 072 demonstrated secretion of a set of proteins typical for white-rot fungi containing both polysaccharide hydrolyzing and oxidizing enzymes. At the same time, on all sawdust-containing media, *P. lycii* LE-BIN 2142 secreted only LPMO and one of its typical electron suppliers—GMC oxidoreductase. This clearly indicates that *P. lycii* LE-BIN 2142 preferentially uses only the oxidative route of polysaccharide degradation. Moreover, this very unusual behavior for white-rot fungi may be typical for *Peniophora* spp. in general.

### 3.3. Secretion of Enzymes Related to Degradation of Lignin

In exoproteomes of both *P. lycii* LE-BIN 2142 and *T. hirsuta* LE-BIN 072, oxidative enzymes related to lignin degradation were the most abundant proteins on all studied media (Figure 4). Although all scientists agreed that enzymatic degradation of such recalcitrant phenolic irregular polymer as lignin can be performed only via oxidative pathways, the exact enzymes involved in initial lignin degradation (i.e., oxidative depolymerization) are still under debate. For a long time, laccases were regarded as the main ligninolytic enzymes of white-rot fungi; however, after the discovery of POD (i.e., lignin, manganese and versatile peroxidases—LiP, MnP and VP, respectively)—in the late 20th century, this had been questioned [37]. Currently, the possession of POD is considered as a necessary and sufficient condition to classify a fungus as white-rot [24,25], and lack of PODs became a hallmark of brown-rot fungi, which are not capable of lignin degradation [38].

The data obtained in the current investigation suggest that for *T. hirsuta* LE-BIN 072, the main secreted enzymes on all media were ligninolytic peroxidases and laccases (Figure 4). The ligninolytic peroxidases were mainly presented by VP2 (secreted on all media) and MnP5 (secreted on all but **GP+Birch** medium) isozymes, and laccases were mainly presented by the LacA isozyme (secreted on all media). Additionally, the MnP6 isozyme was detected on **GP** medium and the LiP9 isozyme on **GP** and **GP+Pine** media.

The secretion of several different isozymes of ligninolytic peroxidases and laccases in response to lignocellulose substrate is typical for white-rot fungi from the Polyporales order, with the exception of the Phanerochaetaceae clade, fungi from which lack laccase genes. Our previous data demonstrated secretion of VP2 and MnP7 isozymes as well as LacA and, to a much lesser extent, LacC isozymes by *T. hirsuta* LE-BIN 072 cultivated on straw-containing medium [39]. The *T. versicolor* strain cultivated by Presley at al. [34] on aspen wafer extract secreted three LiP, six MnP and three laccase isozymes, while the other strain of *T. versicolor* cultivated by del Cerro et al. [35] on poplar-supplemented liquid media secreted two laccase and two MnP isozymes. In the works of Miyauchi et al. and Levasseur et al. [29,30,40] several *Pycnoporus* spp. cultivated on different lignocellulose media differentially secreted several ligninolytic peroxidase and laccase isozymes.

Remarkably, all exoproteomes of *P. lycii* LE-BIN 2142 were characterized by the complete absence of lignolytic peroxidases (Figure 4 and Table 1), while laccases were mainly present by three isozymes—Lac5, Lac7 and Lac8. The Lac5 isoenzyme was secreted on all media, while Lac7 and Lac8 were secreted on all but **GP+Birch** medium. Moreover, on all media, *P. lycii* LE-BIN 2142 predominantly secreted an FAD-binding domain-containing protein provisionally assigned to the AA7 CAZyme family. In accordance with our data, Ma et al. [17] previously showed that *P. incarnata* T-7, cultivated in the presence of poplar wood, and *Peniophora* sp. CBMAI 1063 [16], cultivated in a bioreactor on a nutritionally reach medium supplemented with CuSO_4_, did not secrete ligninolytic type II peroxidases. However, several laccases and, notably, a substantial amount of AA7 CAZyme family proteins were identified in both the mentioned exoproteomes.

The virtual total absence of POD in exoproteomes of *P. lycii* LE-BIN 2142, despite its being a well-known white-rot fungus, suggests its unusual mechanisms of lignin degradation. Although lignin degradation without secretion of POD was documented previously for several typical white-rot fungi such as *Pycnoporus cinnabarinus* [41] and for a newly proposed class of “grey-rot fungi” completely lacking POD in their genomes [24,42,43], the explanation of the precise degradation mechanism is currently absent. Our data suggest that most probably *P. lycii* LE-BIN 2142 degrades lignin using a system of several interconnected oxidative enzymes including laccases, LPMO, AA7 CAZyme family proteins and GMC oxidoreductase, synergetic action of which can produce H_2_O_2_, phenol radicals and other reactive oxygen species with the capability of modifying or partially depolymerizing lignin.

## 4. Materials and Methods

### 4.1. Fungal Strains

The fungal strains *P. lycii* LE-BIN 2142 and *T. hirsuta* LE-BIN 072 were obtained from the Komarov Botanical Institute Basidiomycetes Culture Collection (LE-BIN; St. Petersburg, Russia). The sequences of their ITS1-5.8S rRNA-ITS2 region are available at the NCBI GenBank accessions JX046435.1 and AB158313.1 for *P. lycii* LE-BIN 2142 and *T. hirsuta* LE-BIN 072, respectively. For *T. hirsuta* LE-BIN 072, the Whole Genome Shotgun project had been deposited at DDBJ/ENA/GenBank under the accession PRJNA271118. In the laboratory, both strains were stored on wort-agar slants at 4 °C.

### 4.2. Cultivation on Solid Media

To obtain a starting inoculum, the fungi were cultivated in Petri dishes (Ø 90 mm) containing 20 mL of malt extract-agar (MEA) medium consisting of 1.5% *w*/*v* malt extract (Conda, Madrid, Spain) and 2% *w*/*v* agar (Difco, Kansas City, MO, USA). The cultivation was carried out at 25 °C in the dark until the fungal mycelium had completely overgrown the Petri dish. The inoculum plugs (Ø 7 mm) were seized from the overgrown Petri dishes. All the inoculations were performed by placing the inoculum plugs’ mycelium up in the center of Petri dishes (Ø 90 mm).

To characterize fungal growth on sawdust of different wood species, the fungi were cultivated in Petri dishes (Ø 90 mm) containing 20 mL of MEA medium and 15 g·L^−1^ of either alder, birch or pine sawdust; control cultivations were performed on MEA medium without sawdust. The sawdust was obtained by grinding wood chips on IKA M20 universal mill (IKA, Werke Staufen, Germany) followed by sifting through a 3 mm sieve. The cultivation was carried out at 25 °C in the dark.

To characterize fungal growth on individual wood components, the fungi were cultivated in Petri dishes (Ø 90 mm) containing 2% *w*/*v* agar and either 10 g·L^−1^ of carboxymethyl cellulose (CMC), birch xylan, larch xylan or alkali low sulfonate content lignin (Sigma, St. Louis, MO, USA), or 1 g·L^−1^ of azo-CMC, azo-birch xylan, azo-xyloglucan or azo-arabinoxylan (Megazyme, Wicklow, Ireland); control cultivations were performed on the medium containing only agar. The cultivation was carried out at 25 °C in the dark.

To determine the growth rate of fungal mycelium, the mycelium diameter was daily measured in three directions with a metric rule for 15 days; the obtained measurements (i.e., technical replicates) were averaged. The growth rate was calculated as a slope of growth curve (mycelial diameter vs. time) at the exponential phase of growth.

### 4.3. Cultivation on Semi-Solid Media

To obtain a starting inoculum, the fungi were statically cultivated in 750 mL Erlenmeyer flasks containing 200 mL of glucose–peptone (GP) medium consisting of 3.0 g·L^−1^ peptone, 10.0 g·L^−1^ glucose, 0.6 g·L^−1^ KH_2_PO_4_, 0.4 g·L^−1^ K_2_HPO_4_, 0.5 g·L^−1^ MgSO_4_, 50 mg·L^−1^ MnSO_4_, 1 mg·L^−1^ ZnSO_4_, and 0.5 mg·L^−1^ FeSO_4_. The cultivation was carried out at 28 °C in the dark until the formation of a floating mycelial mat covering the entire surface of the medium. The inoculum was obtained by the disruption of the mycelial mat with ceramic beads at 250 RPM for 15 min. All the inoculations were performed on 25 mL of disrupted mycelium.

To characterize fungal exoproteomes, the semi-solid cultivations were performed in 750 mL Erlenmeyer flasks containing 200 mL of GP medium and 5 g of either alder, birch or pine sawdust; control cultivations were performed on GP medium without sawdust. The sawdust was obtained as described in Section 4.2. Each flask contained a floating nylon mesh disk that supported mycelium growth. The flasks were statically cultivated at 28 °C in the dark; *P. lycii* LE-BIN 2142 was cultivated for 24 days, and *T. hirsuta* LE-BIN 072 was cultivated for 12 days.

### 4.4. Agar Plate Assays of Overall Oxidative and Cellulolytic Activities

Agar plate assays of overall oxidative and cellulolytic activities were performed as previously described in [44]. The inoculum plugs (Ø 7 mm) were seized from the overgrown Petri dishes (see Section 2.2) and placed mycelium up into new Petri dishes (Ø 90 mm) containing 20 mL of the analytical medium consisting of 1% *w*/*v* agar and either 0.1% *w*/*v* 2,2′-azino-bis 3-ethylbenzothiazoline-6-sulfonic acid (ABTS; Sigma, St. Louis, MO, USA) for the overall oxidative activity assay or 1% *w*/*v* cellulose pulp (Sigma, St. Louis, MO, USA) for the overall cellulolytic activity assay. The incubation was carried out for at 25 °C in the dark for 48 h. The overall oxidative activity was evaluated as a diameter of blue colored zones around the plugs, and the overall cellulolytic activity as a diameter of decolorized zones around the plugs that were revealed by water solution of I_2_ in KI (0.5% of I_2_ in 2% KI).

### 4.5. Study of Exoproteomes

The exoproteome extraction, sample preparations, two-dimensional gel electrophoresis (2-DE), MALDI-TOF/TOF MS/MS analysis, and data processing were performed as previously described in [45]. Briefly, the procedure was as follows: (1) samples of mycelium-free cultural broth were filtered (0.45 µm membrane filter) and desalted–concentrated on a Labscale TTF system using the Biomax 5 membrane (Millipore, Burlington, MA, USA); (2) the proteins were precipitated by an equal volume of ice-cold solution of 13.3 mL of TCA and 93 µL of β-mercaptoethanol in 100 mL of acetone; (3) 2-DE was performed on a Protean II xi 2-D Cell system (Bio-Rad, Hercules, CA, USA). The pH gradient on the isoelectrofocusing (IEF) step was from 3 to 10 pH units; (4) the protein spots were visualized by AgNO_3_ staining, photographed using an Infinity1000/26MX gel-documenting system (Vilber Lourmat, Collégien, France) and analyzed by an ImageMaster 2D Platinum v.7 program (GE Healthcare, Buckinghamshire, UK); (5) the proteins were cut from the gel, digested with trypsin and analyzed on an Ultraflex II MALDI TOF/TOF mass spectrometer (Bruker, Bremen, Germany); (6) the analysis of peptide mass fingerprints and MS/MS data was performed using Mascot Server [46]. Additionally, sequences of peptides derived from the MS/MS data were searched with BLAST [47] against an in-house database composed of protein sequences derived from publicly available genomes of *Peniophora* sp. CONTA v1.0 and *Peniophora* sp. v1.0 from the JGI (Joint Genome Institute) portal [48], and *Peniophora* sp. CONT (the same with *Peniophora* sp. v1.0 from the JGI), *Peniophora* sp. CBMAI and *T. hirsuta* LE-BIN 072 from the GeneBank database [49]; (7) the approximate assessment of the relative protein quantities was performed using the spectral counting approach described in [50].

### 4.6. Statistical Data Analysis and Database Search

The annotation of the *T. hirsuta* LE-BIN 072 genome used in this article was derived using Funannotate pipeline v1.5.0 (https://github.com/nextgenusfs/funannotate, accessed on 1 September 2019) and can be found in the Supplementary Materials section (Supplementary Files S1–S4). The additional annotations of carbohydrate-active enzymes (CAZymes) in the *T. hirsuta* LE-BIN 072, *Peniophora* sp. CBMAI, *Peniophora* sp. CONTA v1.0 and *Peniophora* sp. v1.0 genomes using dbCAN2 meta server [51] were also placed in the Supplementary Materials section (Supplementary Table S1).

In addition to the existing genome annotations, all proteins determined in the fungal exoproteomes were analyzed using NCBI’s conserved domain database [52] and SignalP software [53].

All the cultivations were performed at least in triplicate. For the analysis of exoproteomes, cultural broth from biological replicates were pulled together. All statistical comparisons were firstly performed using an ANOVA omnibus F-test. When the omnibus test demonstrated presence of significantly different means *p* < 0.05), the ANOVAs were followed by Tukey’s HSD (honestly significant difference) *post hoc* tests (*p* < 0.05). Whenever appropriate, the data are represented by the mean ± standard deviation (SD).

## 5. Conclusions

The comparison of two white-rot fungi, *P. lycii* LE-BIN 2142 from the Russulales order and *T. hirsuta* LE-BIN 072 from the Polyporales order, demonstrated that although *P. lycii* LE-BIN 2142 showed a growth pattern similar to *T. hirsuta* LE-BIN 072 on various sawdust (i.e., alder, birch and pine) and wood components (i.e., various hemicellulases, cellulose and lignin), these fungi used extremely different enzymatic systems for the degradation of carbohydrates and lignin. Instead of using cellulolytic and hemicellulolytic hydrolytic enzymes, *P. lycii* LE-BIN 2142 primarily relies on oxidative polysaccharide-degrading enzymes such as LPMO and GMC oxidoreductase. Moreover, we hypothesized that oxidative polysaccharide-degrading enzymes together with laccases and previously uncharacterized FAD-binding domain-containing proteins comprise a complex network which provides various oxidative equivalents for modification and depolymerization of lignin. The use of such complex network instead of more conventional POD enzymes makes *P. lycii* LE-BIN 2142 a white-rot fungus with “shades of gray”, adding complexity to the existent wood-degrading fungi classification. Additionally, revealed differences in the enzymatic machineries used by the studied fungi allow, in the future, designing different pure-enzyme-based compositions targeting specific needs of various sectors of the wood processing industry.

## Figures and Tables

**Table 1:** Functional description and secretion conditions for the proteins detected in the exoproteomes of *P. lycii* LE-BIN 2142 and *T. hirsuta* LE-BIN 072 during their semi-solid cultivation on media containing various types of sawdust.

Hit Accession	Hit Species ^1^	Hit Description	EC	CAZY	SignalP	GP ^2^	AL	BR	PN
***Peniophora lycii* LE-BIN 2142**									
**Plant cell wall—Cellulose**									
KZV77634.1	PenSpv1	Lytic polysaccharide monooxygenase	1.14.99.54	AA9	Yes	1	1	1	1
KZV61898.1	PenSpv1	GMC oxidoreductase		AA3_2	No	0	1	1	1
VDB89641.1	PenCBMAI	N-terminal domain of expansins			Yes	0	0	0	1
**Plant cell wall—Hemicellulose**									
KZV62723.1	PenSpv1	endo-*α*-L-arabinanase	3.2.1.55|3.2.1.99	GH43	Yes	1	1	1	1
KZV64181.1	PenSpv1	Esterases and lipases	3.1.1.-	CE15	Yes	0	1	1	0
**Plant cell wall—Pectine**									
KZV68058.1	PenSpv1	Unsaturated glucuronyl hydrolase	3.2.1.-	GH88	Yes	0	1	0	1
**Plant cell wall—Starch**									
KZV59596.1	PenSpv1	Glucoamylase	3.2.1.3	GH15 + CBM20	Yes	0	1	1	1
**Plant cell wall—Lignin**									
KZV72897.1	PenSpv1	Laccase 5^3^	1.10.3.2	AA1_1	Yes	1	1	1	1
KZV76831.1	PenSpv1	Laccase 7	1.10.3.2	AA1_1	Yes	1	1	0	1
KZV67752.1	PenSpv1	Laccase 8	1.10.3.2	AA1_1	Yes	1	1	0	1
KZV69698.1	PenSpv1	Laccase 15	1.10.3.2	AA1_1	Yes	0	1	0	0
KZV66389.1	PenSpv1	Laccase 18	1.10.3.2	AA1_1	Yes	0	1	0	0
KZV64391.1	PenSpv1	FAD-binding domain-containing protein		AA7	Yes	1	1	1	1
KZV64574.1	PenSpv1	FAD-binding domain-containing protein		AA7	Yes	1	1	1	1
KZV63916.1	PenSpv1	FAD-binding domain-containing protein		AA7	Yes	1	1	1	1
**Fungal cell wall**									
KZV74929.1	PenSpv1	*β*-1,3-glucanase; *β*-1,3-glucosidase	3.2.1.39|3.2.1.-	GH128	Yes	1	1	1	1
KZV74469.1	PenSpv1	endo-*β*-1,3(4)-glucanase	3.2.1.6	GH16_1	Yes	1	1	1	1
KZV69996.1	PenSpv1	endo-*β*-1,3(4)-glucanase	3.2.1.6	GH16_1	Yes	1	1	1	1
KZV69997.1	PenSpv1	endo-*β*-1,3(4)-glucanase	3.2.1.6	GH16_1	Yes	1	1	1	1
KZV61773.1	PenSpv1	*β*-1,3-glucanase	3.2.1.39	GH152	Yes	1	1	1	1
KZV69792.1	PenSpv1	*β*-1,3-glucanase	3.2.1.39	GH152	Yes	1	1	1	1
KZV61339.1	PenSpv1	Type II chitinase	3.2.1.14	GH18	Yes	1	1	1	1
KZV67604.1	PenSpv1	*β*-1,3-glucanosyltransglycosylase	2.4.1.-	GH72 + CBM43	Yes	0	1	1	1
KZV73237.1	PenSpv1	*α*-1,3-glucanase	3.2.1.59	GH71	Yes	0	0	1	0
**Other**									
KZV69230.1	PenSpv1	Peptidase S53			No	1	1	1	1
KZV67034.1	PenSpv1	Ricin B lectin			No	1	1	1	0
KZV71566.1	PenSpv1	Lipase class 3			Yes	0	1	1	0
KZV69610.1	PenSpv1	Putative isomerase YbhE			No	0	1	1	0
KZV64666.1	PenSpv1	Cerato-platanin			Yes	0	1	1	0
KZV63020.1	PenSpv1	Glycopeptide			Yes	1	0	0	0
**Hypothetical proteins**									
KZV63574.1	PenSpv1	Hypothetical protein (143 aa)			Yes	1	1	1	1
KZV71782.1	PenSpv1	Hypothetical protein (445 aa)			Yes	1	1	1	1
KZV75687.1	PenSpv1	Hypothetical protein (195 aa)			No	1	1	1	1
KZV75838.1	PenSpv1	Hypothetical protein (390 aa)			Yes	1	1	1	1
KZV66557.1	PenSpv1	Hypothetical protein (269 aa)			Yes	1	1	1	1
KZV74951.1	PenSpv1	Hypothetical protein (143 aa)			Yes	1	1	0	0
VDC00636.1	PenCBMAI	Hypothetical protein (446 aa)			Yes	0	0	1	1
KZV68227.1	PenSpv1	Hypothetical protein (190 aa)			Yes	0	1	0	0
KZV75056.1	PenSpv1	Hypothetical protein (244 aa)			No	0	1	0	0
KZV69008.1	PenSpv1	Hypothetical protein (104 aa)			No	0	1	0	0
KZV75686.1	PenSpv1	Hypothetical protein (187 aa)			Yes	0	0	0	1
***Trametes hirsuta* LE-BIN 072**									
**Plant cell wall—Cellulose**									
FUN_000808	TraHir	Cellulase	3.2.1.4	GH5_5 + CBM1	Yes	0	0	0	1
FUN_005645	TraHir	Cellobiohydrolase I	3.2.1.176	GH6 + CBM1	Yes	0	0	0	1
FUN_000756	TraHir	Cellobiohydrolase II	3.2.1.91	GH7 + CBM1	Yes	0	0	0	1
FUN_010354	TraHir	Cellobiose dehydrogenase	1.1.99.18	AA8 + AA3_1	Yes	0	1	1	1
FUN_000419	TraHir	Lytic polysaccharide monooxygenase	1.14.99.54	AA9	Yes	1	0	0	0
**Plant cell wall—Hemicellulose**									
FUN_001307	TraHir	*β*-mannosidase	3.2.1.25	GH2	Yes	0	1	0	1
**Plant cell wall—Pectine**									
FUN_002751	TraHir	Polygalacturonase	3.2.1.15	GH28	Yes	1	1	1	1
FUN_008017	TraHir	Tannase; feruloyl esterase	3.1.1.20|3.1.1.73	CE1	Yes	1	0	0	0
**Plant cell wall—Starch**									
FUN_006083	TraHir	Glucoamylase	3.2.1.3	GH15 + CBM20	Yes	1	1	1	1
FUN_010348	TraHir	Maltogenic Amylase	3.2.1.1|3.2.1.98|3.2.1.116	GH13_32 + CBM20	Yes	0	0	1	1
**Plant cell wall—Lignin**									
FUN_001030	TraHir	Laccase A	1.10.3.2	AA1_1	Yes	1	1	1	1
FUN_010573	TraHir	Laccase C	1.10.3.2	AA1_1	Yes	0	0	1	0
FUN_007604	TraHir	VP2 (POD17)	1.11.1.16	AA2	Yes	1	1	1	1
FUN_002850	TraHir	MnP5 (POD05)	1.11.1.13	AA2	Yes	1	1	0	1
FUN_007002	TraHir	LiP9 (POD18)	1.11.1.14	AA2	Yes	1	0	0	1
FUN_007983	TraHir	MnP6 (POD06)	1.11.1.13	AA2	Yes	1	0	0	0
FUN_010480	TraHir	Glyoxal oxidase GLOX	1.2.3.15	AA5_1	Yes	1	1	1	1
FUN_001506	TraHir	Glyoxal oxidase GLOX	1.2.3.15	AA5_1	Yes	1	1	1	1
**Fungal cell wall**									
FUN_000340	TraHir	*β*-1,3-glucanosyltransglycosylase	2.4.1.-	GH72 + CBM43	No	0	1	1	1
FUN_002148	TraHir	endo-*β*-1,3(4)-glucanase		GH16_1	Yes	1	1	0	1
FUN_010090	TraHir	*β*-1,3-glucanase	3.2.1.39	GH152	Yes	0	1	0	0
**Proteases**									
FUN_008047	TraHir	Peptidase S53			Yes	1	1	1	1
FUN_006633	TraHir	Peptidase S53			Yes	1	1	1	1
FUN_009590	TraHir	Peptidase S10			No	1	1	1	1
FUN_006927	TraHir	Peptidase S8			Yes	1	1	0	1
FUN_001546	TraHir	Metallopeptidase M36			Yes	0	1	0	1
FUN_006898	TraHir	Aspartyl (acid) protease			Yes	0	0	1	1
FUN_009658	TraHir	Protease family A1			Yes	0	0	1	1
FUN_002544	TraHir	Protease family A1			Yes	0	0	0	1
**Other**									
FUN_002582	TraHir	Lipase/esterase			No	0	1	1	1
FUN_002005	TraHir	Fungal phospholipase B			Yes	0	1	1	1
FUN_010217	TraHir	MBL-fold metallo-hydrolase			Yes	0	1	1	1
FUN_010277	TraHir	Putative isomerase YbhE			No	1	1	0	1
FUN_006612	TraHir	*α*,*α*-trehalase	3.2.1.28	GH37	Yes	1	0	1	1
FUN_010625	TraHir	Lytic murein transglycosylase D			Yes	0	0	1	1
FUN_003015	TraHir	Metal-dependent hydrolase			Yes	0	0	1	1
FUN_007614	TraHir	Ferritin-like superfamily			No	0	0	1	1
FUN_000583	TraHir	Polysaccharide lyase	4.2.2.-	PL8_4	Yes	1	0	0	0
FUN_000786	TraHir	Alginate lyase		PL35	No	1	0	0	0
FUN_000797	TraHir	Alginate lyase		PL35	No	1	0	0	0
FUN_002699	TraHir	Amidase			Yes	0	0	0	1
**Hypothetical proteins**									
FUN_004762	TraHir	Hypothetical protein (165 aa)			Yes	1	1	1	1
FUN_009768	TraHir	Hypothetical protein (385 aa)			Yes	0	1	1	1
FUN_000138	TraHir	Hypothetical protein (641 aa)			No	0	0	1	1
FUN_007129	TraHir	Hypothetical protein (139 aa)			Yes	0	0	1	1
FUN_006467	TraHir	Hypothetical protein (173 aa)			Yes	0	0	0	1
FUN_006175	TraHir	Hypothetical protein (145 aa)			Yes	0	1	0	0
FUN_000212	TraHir	Hypothetical protein (166 aa)			Yes	1	0	0	0

^1^ PenSpv1—*Peniophora* sp. v1.0; PenCBMAI—*Peniophora* sp. CBMAI; TraHir—*T. hirsuta* LE-BIN 072. ^2^ GP—control **GP** medium; AL—**GP+Alder** medium; BR—**GP+Birch** medium; PN—**GP+Pine** medium. ^3^ Naming of laccase and POD isozymes are in accordance with [19,20] for *T. hirsuta* LE-BIN 072 and [21] for *P. lycii* LE-BIN 2142.

## Data Availability

The data presented in this study are available in the article and Supplementary Materials.

## Supplementary Materials

The supporting information can be downloaded at: https://www.mdpi.com/article/10.3390/ijms231810322/s1.
