# Supplementary material for: Comparative Analysis of Peniophora lycii and Trametes hirsuta Exoproteomes Demonstrates “Shades of Gray” in the Concept of White-Rotting Fungi
Source: Int J Mol Sci. 2022 Sep 7;23(18):10322. doi: 10.3390/ijms231810322 (PMC9499651; doi:10.3390/ijms231810322)
Supplement: Supplementary file 1 [file ijms-23-10322-s001.zip › Supplementary Materials/3)Supplementary_Figures/1)Supplementary FigureS1-Growth_and_activities_on_sawdust.pdf]

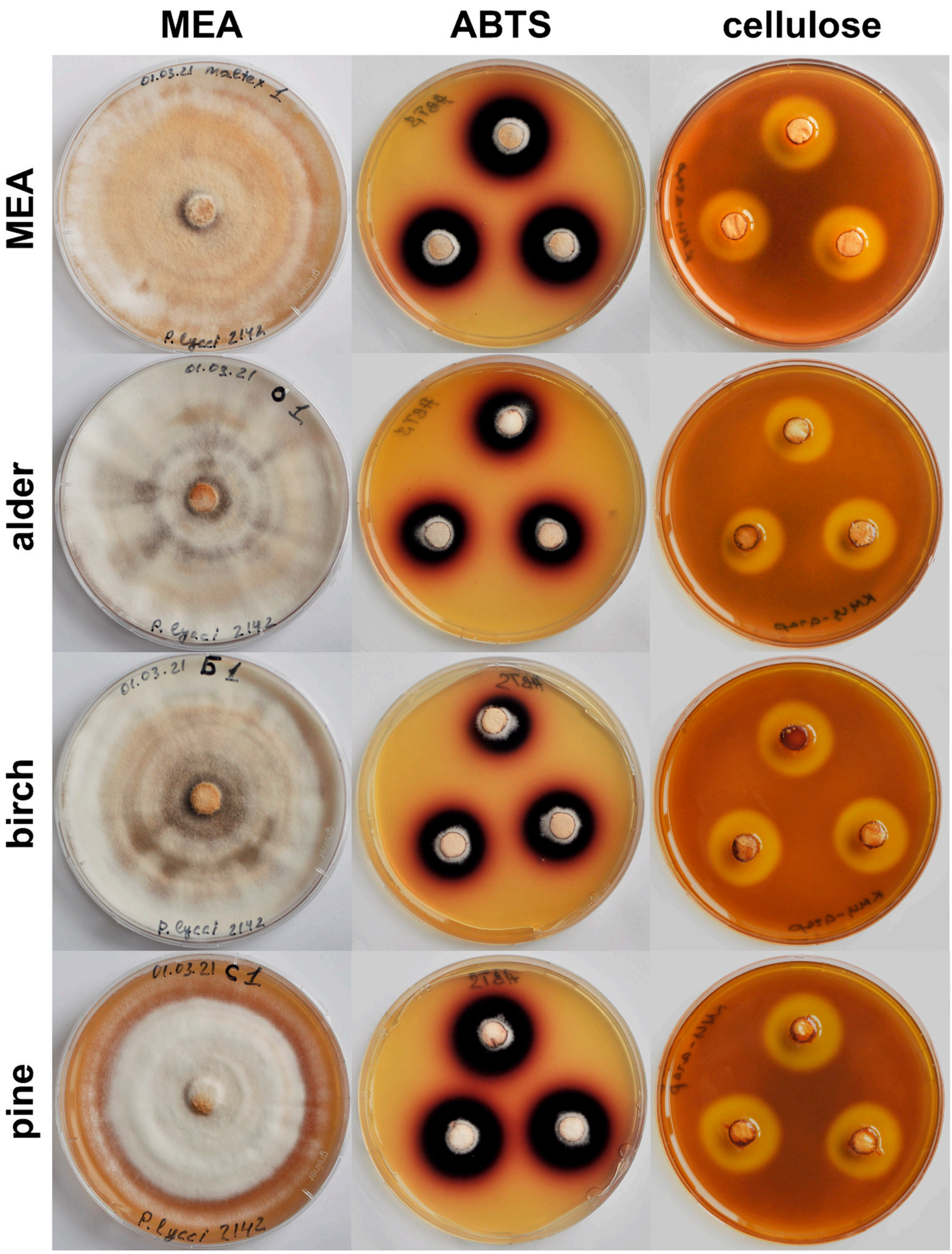

(A)

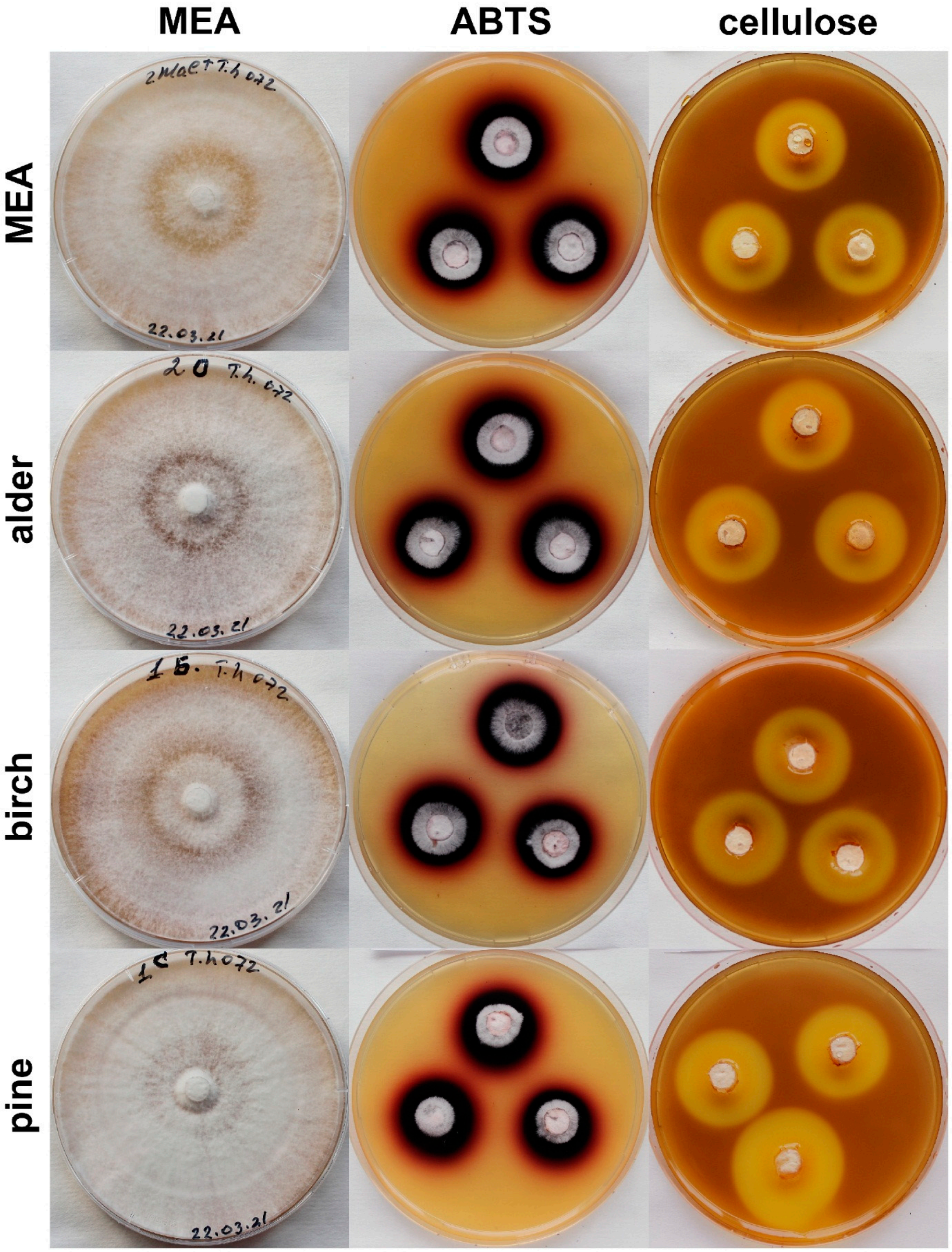

(B)

Figure S1. (**A**) Mycelium appearance as well as agar plate assays of overall oxidation (ABTS) and cellulolytic (cellulose pulp) activities of *Peniophora lycii* LE-BIN 2142 during its cultivation on MEA media containing various types of sawdust – alder, birch and pine. (**B**) Mycelium appearance as well as agar plate assays of overall oxidation (ABTS) and cellulolytic (cellulose pulp) activities of *Trametes hirsuta* LE-BIN 072 during its cultivation on MEA media containing various types of sawdust – alder, birch and pine.
