# Supplementary material for: Comparative Analysis of Peniophora lycii and Trametes hirsuta Exoproteomes Demonstrates “Shades of Gray” in the Concept of White-Rotting Fungi
Source: Int J Mol Sci. 2022 Sep 7;23(18):10322. doi: 10.3390/ijms231810322 (PMC9499651; doi:10.3390/ijms231810322)
Supplement: Supplementary file 1 [file ijms-23-10322-s001.zip › Supplementary Materials/3)Supplementary_Figures/3)Supplementary FigureS3-2D_gels.pdf]

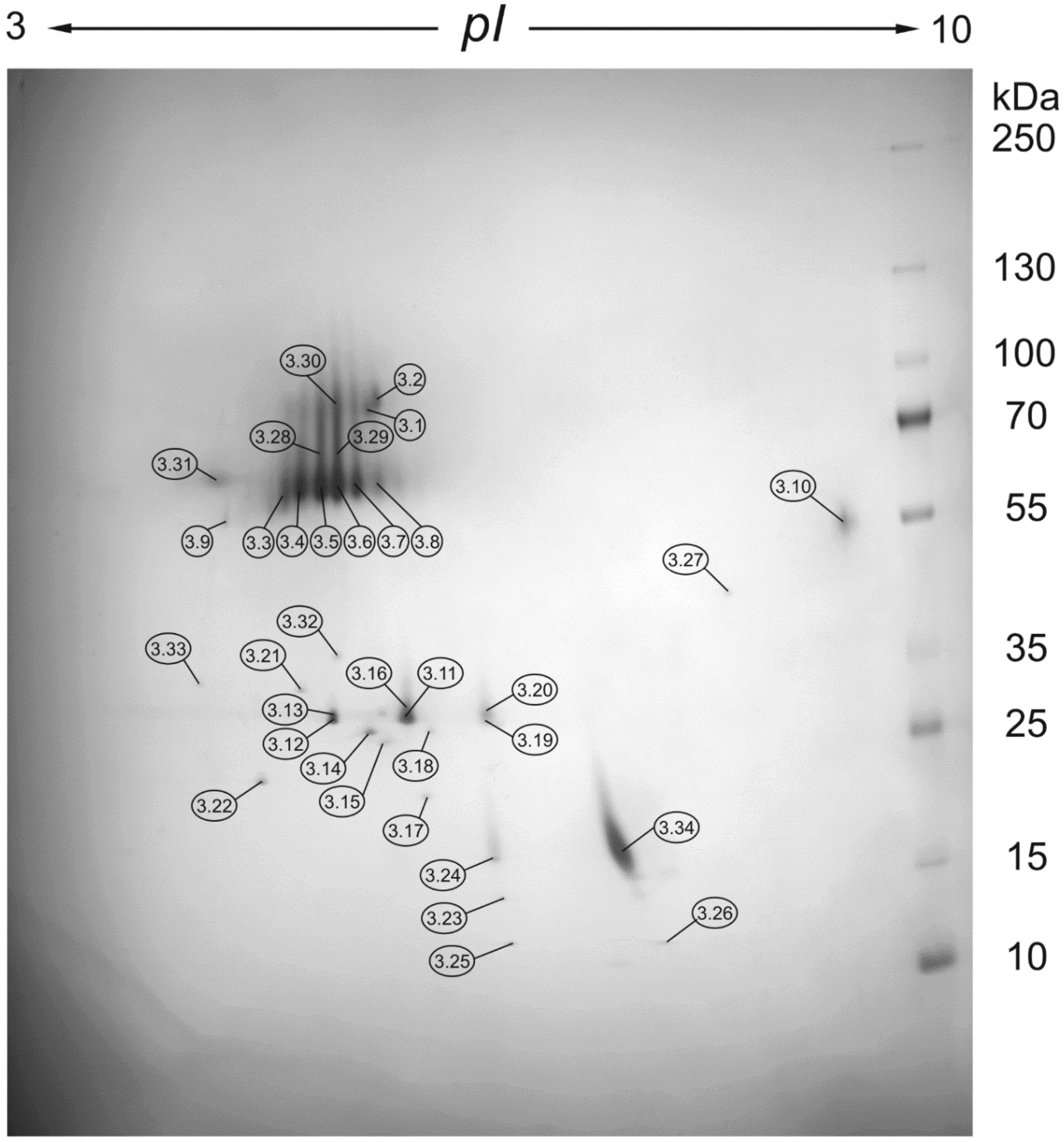
3
pI
10
kDa
250
130
100
70
55
35
25
15
10
3.30
3.2
3.1
3.28
3.29
3.31
3.10
3.9
3.3
3.4
3.5
3.6
3.7
3.8
3.27
3.32
3.33
3.21
3.16
3.11
3.20
3.13
3.19
3.12
3.14
3.18
3.22
3.15
3.17
3.34
3.24
3.23
3.26
3.25

(A)

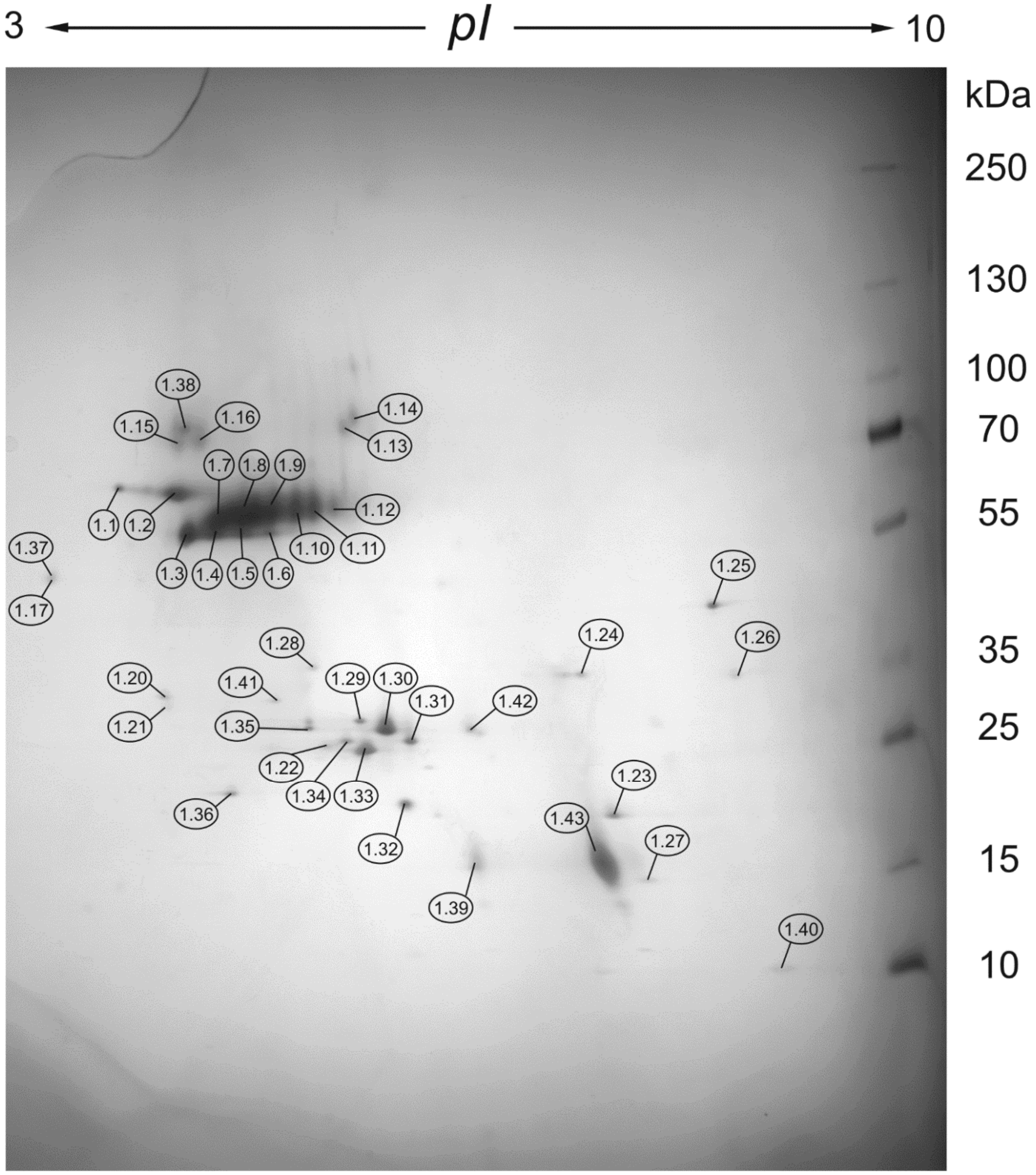
3
pI
10
kDa
250
130
100
70
55
35
25
15
10
1.1
1.2
1.3
1.4
1.5
1.6
1.7
1.8
1.9
1.10
1.11
1.12
1.13
1.14
1.15
1.16
1.17
1.20
1.21
1.22
1.23
1.24
1.25
1.26
1.27
1.28
1.29
1.30
1.31
1.32
1.33
1.34
1.35
1.36
1.37
1.38
1.39
1.40
1.41
1.42
1.43

(B)

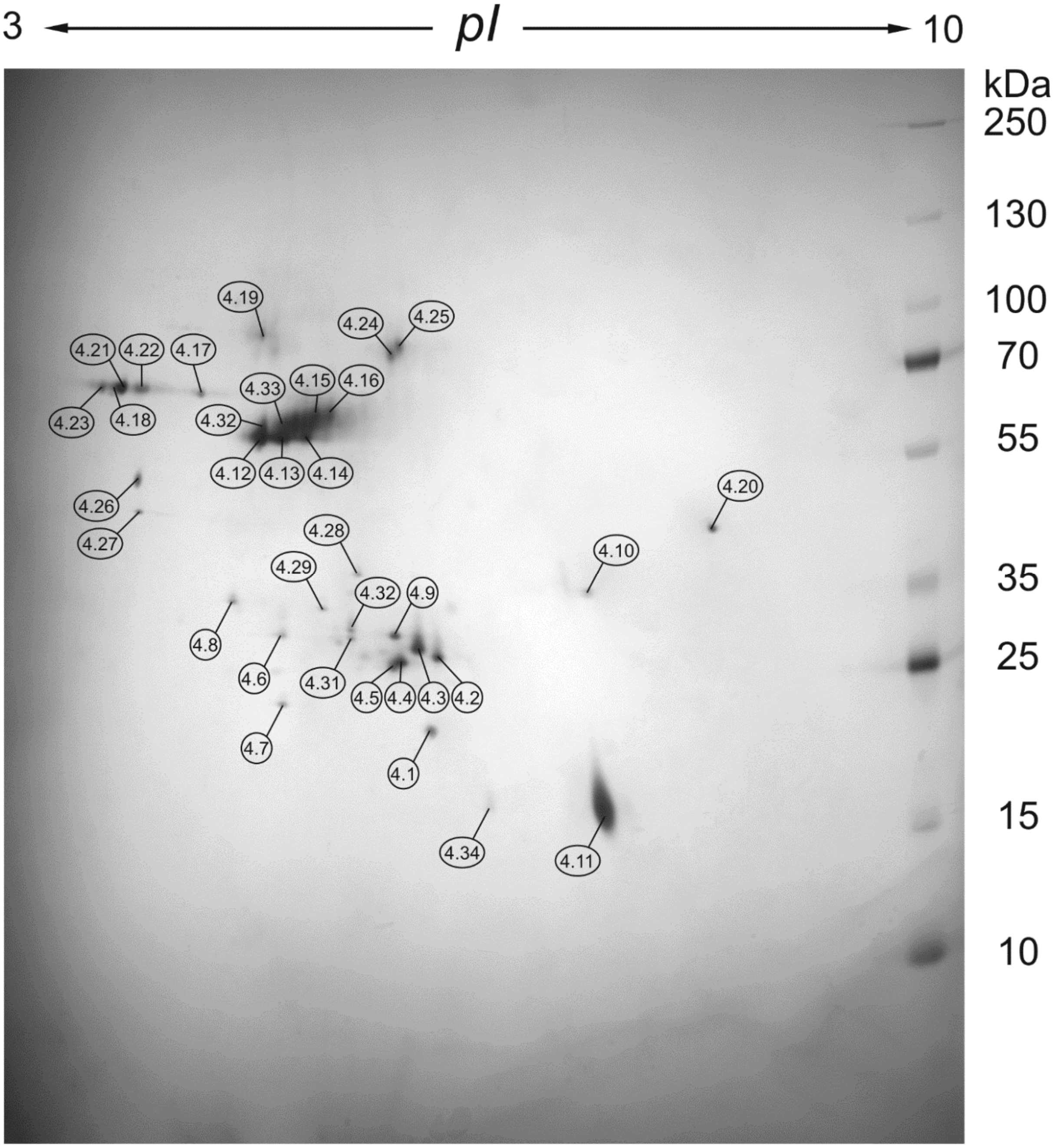
3
pI
10
kDa
250
130
100
70
55
35
25
15
10
4.19
4.24
4.25
4.21
4.22
4.17
4.33
4.15
4.16
4.23
4.18
4.32
4.12
4.13
4.14
4.20
4.26
4.27
4.28
4.10
4.29
4.32
4.9
4.8
4.6
4.31
4.5
4.4
4.3
4.2
4.7
4.1
4.34
4.11

(C)

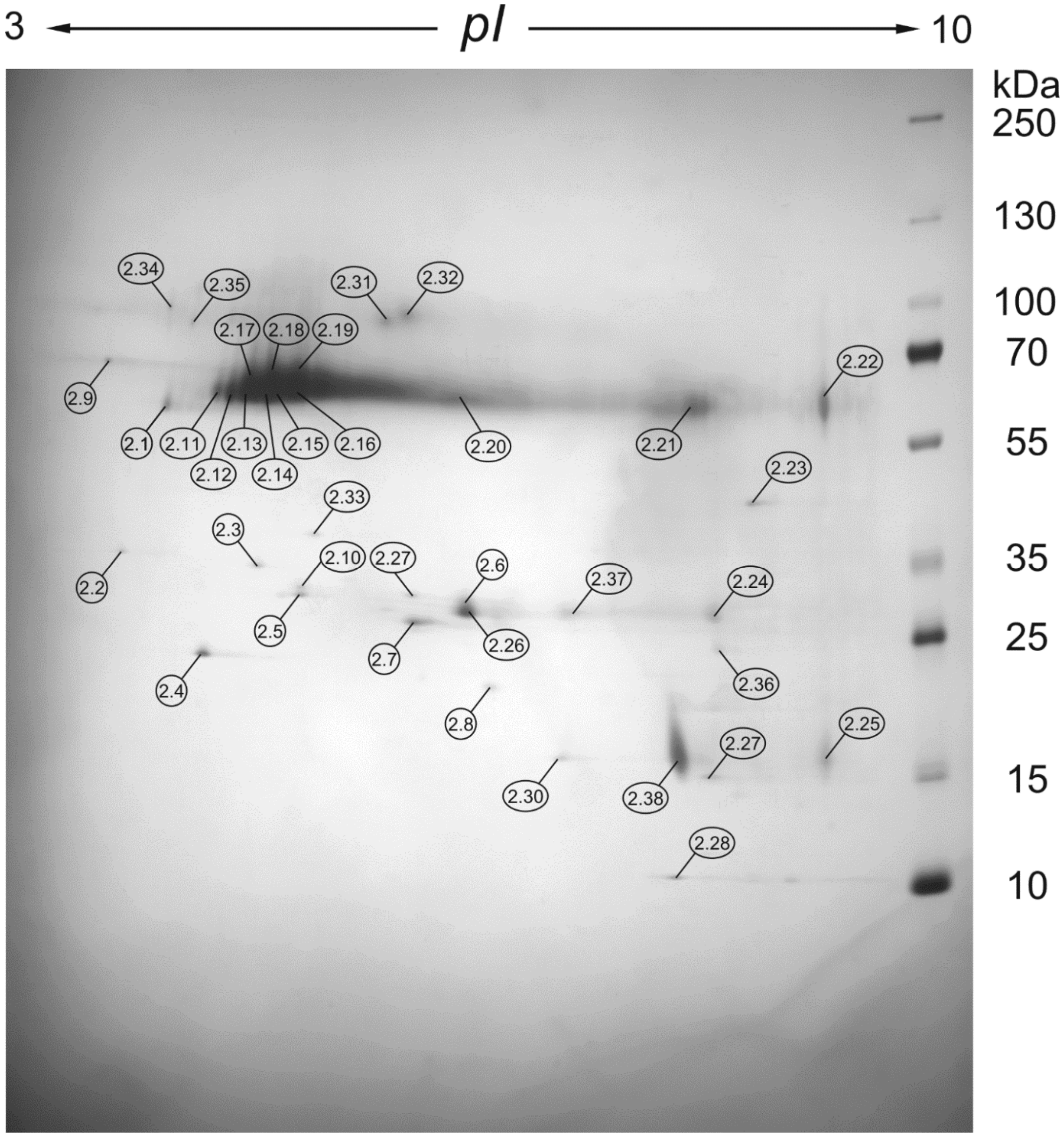

(D)

Figure S3. (**A**) Exoproteome of *Peniophora lycii* LE-BIN 2142 cultivated on control glucose-peptone (GP) medium. For the data on MALDI TOF/TOF MS/MS analysis of the highlighted protein spots, please, refer to Supplementary Table S2. (**B**) Exoproteome of *Peniophora lycii* LE-BIN 2142 cultivated on GP medium containing sawdust of alder. For the data on MALDI TOF/TOF MS/MS analysis of the highlighted protein spots, please, refer to Supplementary Table S2. (**C**) Exoproteome of *Peniophora lycii* LE-BIN 2142 cultivated on GP medium containing sawdust of birch. For the data on MALDI TOF/TOF MS/MS analysis of the highlighted protein spots, please, refer to Supplementary Table S2. (**D**) Exoproteome of *Peniophora lycii* LE-BIN 2142 cultivated on GP medium containing sawdust of pine. For the data on MALDI TOF/TOF MS/MS analysis of the highlighted protein spots, please, refer to Supplementary Table S2.
