# Supplementary figures and images for: Comparative Analysis of Peniophora lycii and Trametes hirsuta Exoproteomes Demonstrates “Shades of Gray” in the Concept of White-Rotting Fungi
Source: Int J Mol Sci. 2022 Sep 7;23(18):10322. doi: 10.3390/ijms231810322 (PMC9499651; doi:10.3390/ijms231810322)

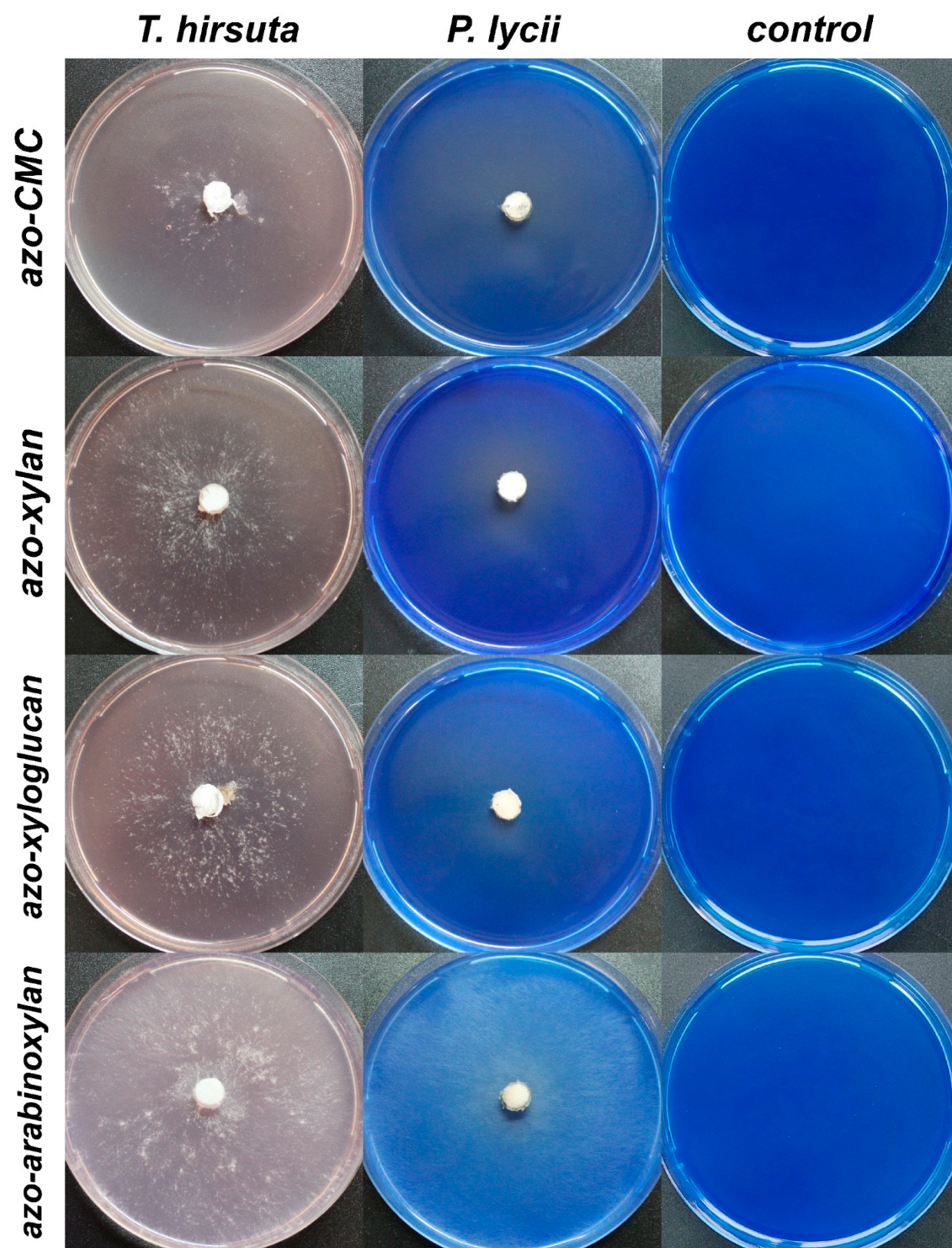

Figure S2. Growth of *Peniophora lycii* LE-BIN 2142 and *Trametes hirsuta* LE-BIN 072 on azo-polysaccharides.

Supplement: Supplementary file 1 [file ijms-23-10322-s001.zip › Supplementary Materials/3)Supplementary_Figures/2)Supplementary FigureS2-Growth_on_azo-polysaccharides.pdf]
